# Primary Sino-nasal Neuroendocrine Carcinoma: A Rare Tumor

**DOI:** 10.7759/cureus.4144

**Published:** 2019-02-27

**Authors:** Shaqul Qamar Wani, Ishtiyaq A Dar, Talib Khan, Mohammad M Lone

**Affiliations:** 1 Radiation Oncology, Sher I Kashmir Institute of Medical Sciences, Srinagar, IND; 2 Anesthesiology, Sher I Kashmir Institute of Medical Sciences, Srinagar, IND

**Keywords:** primary sino-nasal neuroendocrine carcinomas, concurrent chemoradiation, chemotherapy, radiotherapy, radiological resolution, small cell neuroendocrine carcinoma

## Abstract

Primary sino-nasal neuroendocrine carcinomas (PSNNECs) are rare, with a wide spectrum of histological differentiation. Advanced tumors may invade the surrounding structures, such as the skull base, orbit, or brain. Here, we present a rare case of PSNNEC and its management by concurrent chemoradiation (CCRT) followed by adjuvant chemotherapy with the radiological resolution of the tumor mass.

## Introduction

The sino-nasal tract (SNT) is the location of a diversity of malignant tumors among which squamous cell carcinoma is the most common, followed by adenocarcinoma, malignant lymphoma, sino-nasal undifferentiated carcinoma, malignant melanoma, and olfactory neuroblastoma, due to the anatomic complexity and tissue variability [1]. Malignancies of the SNT have shown dramatic improvements in survival, due to the improvements in diagnosis and treatment [1-2].

Malignant SNT tumors are rare in most parts of the world [3-4] and comprise less than 1% of all neoplasms and 3% of the upper aerodigestive tract [5]. However, neuroendocrine carcinomas (NEC) are rare with a wide spectrum of histological differentiation and are classified into well-differentiated (typical carcinoid), moderately differentiated (atypical carcinoid), and poorly differentiated (small and non-small cell types), with the latter one being extremely rare and carrying a poor prognosis due to its aggressive nature with a high potential for recurrence and distant metastasis (DM) regardless of multimodal treatment [6-7]. Keeping in mind the rarity of PSNNEC (small cell type) and the lack of definitive treatment guidelines, we present a case of PSNNEC (small cell type) and its management by concurrent chemo-radiotherapy (CCRT) with optimal response and emphasis in the literature review.

## Case presentation

**Figure 1:**
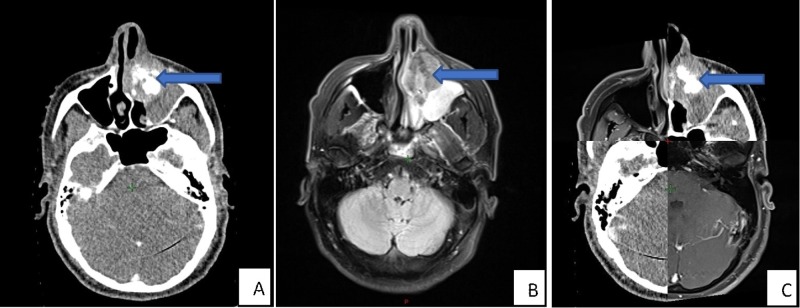
Pre-treatment imaging A – CT: computed tomography, B – MRI: magnetic resonance imaging, and C – CT/MRI fusion scans showing the left primary sino-nasal neuroendocrine carcinoma (PSNNEC) by blue arrowheads

**Figure 2:**
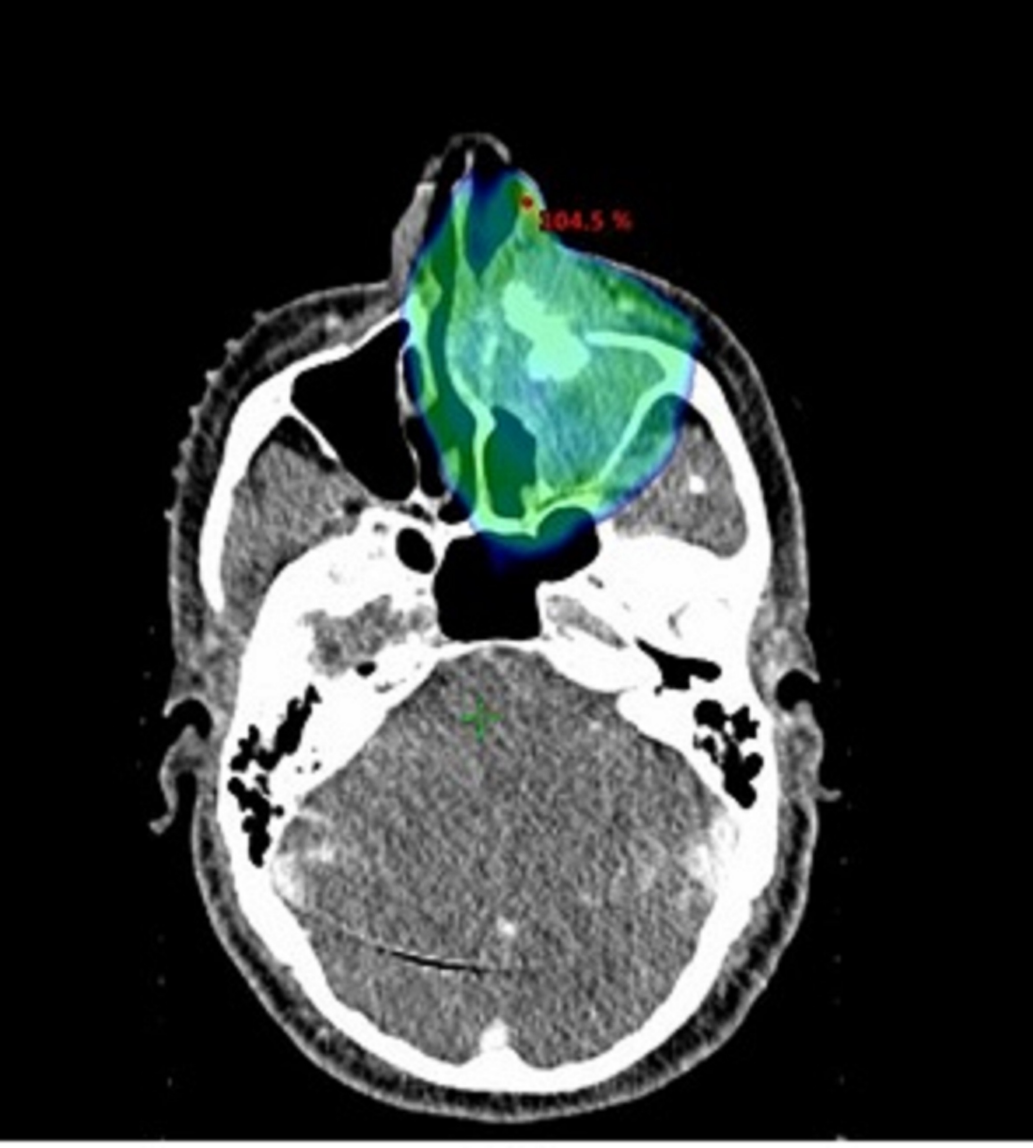
Dose distribution in color wash of the left primary sino-nasal neuroendocrine carcinomas for IMRT planning PSNNEC: primary sino-nasal neuroendocrine carcinoma; IMRT: intensity modulated radiotherapy

**Figure 3:**
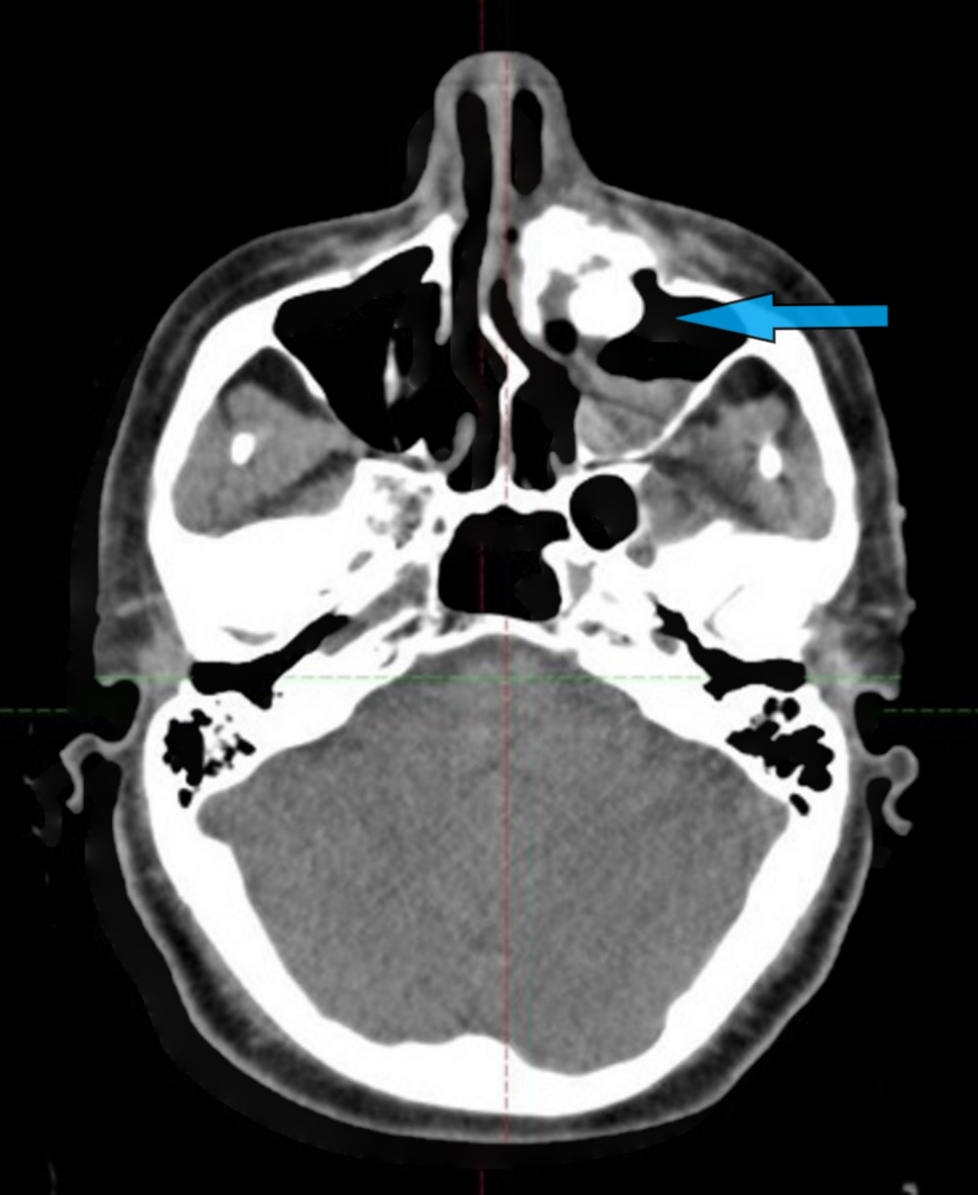
CT evaluation of response at the 5th week of concurrent CCRT of left PSNNECs shown by arrowhead as the optimal radiological resolution CT: computed tomography; CCRT: chemoradiotherapy; PSNNEC: primary sino-nasal neuroendocrine carcinoma

A 45-year-old Asian male presented with a history of nasal bleeding from the left nostril, watering of the left eye, and nasal obstruction (on and off); examination revealed a mass in the left nasal cavity. Computed tomography (CT) and magnetic resonance imaging (MRI) scans revealed a heterogeneous soft tissue attenuation mass in the left anterior nasal cavity, causing the erosion of the medial wall of the left maxillary sinus, showing irregular speculated calcification with a small, extra-osseous soft tissue component in the anterior deep subcutaneous tissue of the cheek, causing the blockage of the left osteomeatal complex and the narrowing of the left inferior meatus with resultant soft tissue attenuation (Figures 1A-1B). Biopsy revealed small cell neuroendocrine carcinoma (SCNEC) strongly positive for cytokeratin (CK) and epithelial membrane antigen (EMA), moderately positive for CD-56 and neuron-specific enolase (NSE) and negative for p-63, CK-5/6, synaptophysin, chromogranin A, desmin, and p-40. The patient had no evidence of distant metastasis and received CCRT with cisplatin and etoposide along with a total radiotherapy (RT) dose of 60 Gy in 30 fractions, delivered by the intensity modulated radiotherapy (IMRT) technique. Target delineation was done after a CT-MRI fusion scan (Figure 1C) and the target coverage (color wash) was between 95% and 107% of the prescribed dose. The clinical target volume (CTV) high was kept equal to the gross tumor volume (GTV) plus a margin of 7 mm (GTV+7 mm) and the planning target volume (PTV) high was kept equal to the CTV high plus a margin of 5 mm (CTV high+5 mm) (Figure 2). The patient also received concurrent cisplatin 75 mg/m^2^ on Day 1 and etoposide 100 mg/m^2^ on Days 1 to 3 (every three-weekly cycle). The CT scan revealed an optimal response at Week 5 of RT (Figure 3). Presently, the patient is on the adjuvant chemotherapy protocol and is planned for three more cycles of chemotherapy. The patient, at present, is symptomatically better and continues regular follow-up.

## Discussion

PSNNEC was first proposed by Silva et al. in 1982 [8] and is an uncommon tumor with no definite demographic preference, such as sex, race, or geography and no correlation with smoking or radiation. The mean age of presentation is around 49 years and ranges from 26 to 77 years. SCNEC commonly arises in the nasal cavity and often extends into the adjacent sinuses (maxillary or ethmoid). The primary involvement of the maxillary or ethmoid sinuses without nasal extension can be seen in approximately 45% of cases. Advanced tumors may invade the skull base, orbit, or brain [6-8].

The limited number of cases published, difficulties of diagnosis, and heterogeneity of treatment approaches hamper evaluating the ideal treatment strategy [9-11]. Predictors of poor outcomes were patients with orbital involvement and tumor originating outside the nasal cavity. Surgery followed by adjuvant RT was the primary approach to treat small cell tumors in the 1980s. Perez-Ordonez et al., have emphasized the use of combined-modality therapy for these tumors [9]. Neoadjuvant treatment in the form of chemotherapy followed by RT showed promising results, as proposed by Fitzek et al. [10] and Bhattacharyya et al. [12]. A similar treatment protocol was applied in bulky or unresectable tumors by Babin et al. [11], which was also based on the outcome of the 35th Congress of the French Cervico-Faciale Carcinologic Society, France, which revealed complete response to neoadjuvant chemotherapy correlated with improved survival at three years. Given the high incidence of distant failure and the chemosensitivity of NEC, neoadjuvant chemotherapy followed by either CCRT or surgery and postoperative RT is a promising strategy. In patients with advanced tumors, CCRT has shown a promising role besides the resolution of paraneoplastic symptoms like the syndrome of inappropriate antidiuretic hormone (ADH) secretion (SIADH) as shown by Vasan et al. [13]. The role of proton therapy was also established in addition to CCRT in preserving the visual functions in case of olfactory NEC [10]. However, if the margins of the tumor are clear, surgery is also an option in the management of PSNNEC, as suggested by Sirsath et al. [6] and Faisal M et al. [14].

With regards to the treatment protocol in our patient, CCRT has shown a good response in terms of radiological as well as subjective symptomatic control, hence, we recommend that such patients should be subjected to the upfront CCRT rather than subjected to mutilating surgeries, as described in the literature. This report should open the front for future randomized controlled trials to show the beneficial effect of CCRT over surgery.

## Conclusions

Neuroendocrine tumors of the sino-nasal tract are locally recurrent and destructive and a multimodality treatment approach is needed for its management. Neoadjuvant treatment in the form of concurrent chemoradiation followed by adjuvant chemotherapy should be the standard protocol because of the chemoradiosensivity. Surgery, being mutilating, should be the last resort if patients do not show a response to the CCRT protocol.
